# Extracellular Vesicles from BMSCs Prevent Glucocorticoid-Induced BMECs Injury by Regulating Autophagy via the PI3K/Akt/mTOR Pathway

**DOI:** 10.3390/cells11132104

**Published:** 2022-07-03

**Authors:** Jinhui Ma, Mengran Shen, Debo Yue, Weiguo Wang, Fuqiang Gao, Bailiang Wang

**Affiliations:** 1 Department of Orthopaedic Surgery, Center for Osteonecrosis and Joint Preserving & Reconstruction, China-Japan Friendship Hospital, Beijing 100029, China; majinhui@zryhyy.com.cn (J.M.); yuedebo@zryhyy.com.cn (D.Y.); wangweiguo@zryhyy.com.cn (W.W.); 2 Department of Orthopaedic Surgery, Peking University China-Japan Friendship School of Clinical Medicine, Beijing 100029, China; 2111210577@stu.pku.edu.cn

**Keywords:** autophagy, extracellular vesicles, PI3K/Akt/mTOR pathway, bone marrow mesenchymal stem cells, bone microvascular endothelial cells

## Abstract

Osteonecrosis of the femoral head (ONFH) is a common clinical disease with a high disability rate. Injury of bone microvascular endothelial cells (BMECs) caused by glucocorticoid administration is one of the important causes of ONFH, and there is currently a lack of effective clinical treatments. Extracellular vesicles derived from bone stem cells (BMSC-EVs) can prevent ONFH by promoting angiogenesis and can inhibit cell apoptosis by regulating autophagy via the PI3K/Akt/mTOR signaling pathway. The present study aimed to investigate the effect of extracellular vesicles derived from bone marrow stem cells (BMSC) on a glucocorticoid-induced injury of BMECs and possible mechanisms. We found that BMSC-EVs attenuated glucocorticoid-induced viability, angiogenesis capacity injury, and the apoptosis of BMECs. BMSC-EVs increased the LC3 level, but decreased p62 (an autophagy protein receptor) expression, suggesting that BMSC-Exos activated autophagy in glucocorticoid-treated BMECs. The protective effects of BMSC-EVs on the glucocorticoid-induced injury of BMECs was mimicked by a known stimulator of autophagy (rapamycin) and could be enhanced by co-treatment with an autophagy inhibitor (LY294002). BMSC-EVs also suppressed the PI3K/Akt/mTOR signaling pathway, which regulates cell autophagy, in glucocorticoid-treated BMECs. In conclusion, the results indicate that BMSC-EVs prevent the glucocorticoid-induced injury of BMECs by regulating autophagy via the PI3K/Akt/mTOR pathway.

## 1. Introduction

Osteonecrosis of the femoral head (ONFH) is the death of bone (including bone cells, bone marrow hematopoietic cells, and fat cells) due to various factors [1]. The pathogenesis of non-traumatic ONFH is still unclear, but may be attributed to altered lipid metabolism/fat emboli, cell and bone death, increased mechanical stress, elevated intracortical pressure, and bone remodeling imbalance [2,3]. These factors diminish femoral blood perfusion through common pathologic mechanisms, including vascular endothelial damage and microvascular thrombosis. Bone microvascular endothelial cells (BMECs) are highly active endocrine cells, which comprise a monolayer structure attached to the inner wall of bone and form bone microvessels. Reduced numbers and impaired function of endothelial progenitor cells have been associated with an increased risk of ONFH [4]. An injury to BMECs caused by glucocorticoid could result in local blood hypercoagulation, microvascular thrombosis, and vascular occlusion, leading to necrosis of the femoral head in the dominant area [5,6]. A large number of reactive oxygen species produced by the injured BMECs could reduce the synthesis of vasodilator substances and further aggravate the injury of BMECs, eventually leading to necrosis of bone cells and bone marrow [7]. Promoting angiogenesis and maintaining vascular permeability of the femoral head is of great significance for the prevention and treatment of ONFH. Therefore, the molecular mechanism of bone microcirculation disorder in the femoral head caused by glucocorticoid-induced damage of BMECs must be further studied.

In recent years, the influence of autophagy on endothelial cells has attracted great interest [8]. Autophagy is a highly conserved biological phenomenon characterized by the formation of double-membrane vesicles called autophagosomes, and the subsequent engulfment and delivery of various cellular components (proteins, organelles, and invading pathogens) to lysosomes for degradation and material recycling [9,10]. The phosphoinositide 3-kinase/protein kinase B/mammalian target of the rapamycin (PI3K/Akt/mTOR) signaling pathway is a prototypic survival pathway that plays a central role in diverse cellular functions, including proliferation, growth, survival, metabolism, and autophagy [11,12]. As a downstream effector of the PI3K/Akt pathway, mTOR activity is the key to autophagosome formation and maturation. The mTOR, a conserved serine/threonine kinase, can integrate various signaling pathways associated with growth factors, stress, and nutrients to promote cell survival and inhibit autophagy [13]. The study conducted by An Y et al. proved that autophagy determines the therapeutic effect of MSCs in cutaneous wound healing through the promotion of endothelial cell angiogenesis, and further revealed that autophagy enhanced the vascular endothelial growth factor secretion from MSCs to promote the angiogenesis of ECs by directly phosphorylating ERK1/2 [14]. In the study by Liao Y et al., Western blot and immunofluorescence staining results showed that the LC3-II/LC3-I ratio and the group of Beclin-1 (autophagy-related proteins) in dexamethasone-induced bone-marrow-derived endothelial progenitor cells (BM-EPCs) gradually decreased from the 12 h time point to the 24 h time point, reaching the lowest level at the 48 h time point; dexamethasone could inhibit autophagy levels in EPCs, and pravastatin could ameliorate ONFH by upregulating the autophagy activity in (BM-EPCs) [15]. Therefore, modulating autophagy in ECs may also be a potential target for the treatment of ONFH.

Recent studies have demonstrated that the autophagy in vascular ECs can be regulated by a range of biological factors and chemical compounds, and may have significant impacts on the fate of ECs. Meanwhile, a previous study suggested that EVs secreted by induced pluripotent stem-cell-derived mesenchymal stem cells significantly enhanced the proliferation, migration, and tube-forming capacities of ECs in vitro by activating the PI3K/Akt signaling pathway [16]. EVs are a class of membrane-bound vesicles with a diameter of 30 to 2000 nm, depending on their origin, that are involved in delivering functional biochemicals, including cytokines, proteins, lipids, and RNAs (mRNAs and miRNAs) into the target cell to stimulate a particular biological function. As determined by their biogenesis, the three main classes of EVs are exosomes, microvesicles, and apoptotic bodies [17,18,19]. The transplantation of EVs has been confirmed to exert similar therapeutic effects to direct stem cell transplantation in tissue repair. Some studies found that the transplantation of EVs secreted by human-induced pluripotent stem-cell-derived mesenchymal stem cells could promote angiogenesis of ischemic tissue in limb ischemia and skin defects that might be useful for other ischemic diseases, including ONFH [20,21]. Some studies have demonstrated the potential relationship of the PI3K/Akt pathway and stem cells in the biological activity of vascular endothelial cells [22]. It was reported that endothelial autophagy could be inhabited via the PI3K/Akt/mTOR signaling pathway, which could contribute to endothelial cell dysfunction. These results suggests that MSC-EVs can regulate ECs’ autophagy via the PI3K/Akt/mTOR signaling pathway, affecting the biological function of ECs. Since exosomes are important functional products of MSCs, the biological function of ECs affected by EVs might be associated with endothelial autophagy via the PI3K/Akt/mTOR signaling pathway. Based on these studies, we hypothesize that the transplantation of MSC-EVs might prevent the progression of ONFH, and the PI3K/Akt/mTOR pathway might be involved in the autophagy of BMECs triggered by MSC-EVs.

## 2. Materials and Methods

### 2.1. BMECs Isolation, Identification and Culture

The human femoral head was obtained from patients undergoing total hip arthroplasty to generate BMECs after obtaining informed consent from patients. Cancellous bone of the femoral head was made into small bone fragments, then placed in a 50 mL centrifuge tube containing serum-free Dulbecco’s Modified Eagle’s Medium (DMEM; Gibco, Grand Island, NY, USA). The centrifuge tube was oscillated every 5 min. We then added 0.1% trypsin −0.1% EDTA (Procell, Wuhan, China), and digested it in a water bath at 37 °C for 5 min. After digestion, the liquid was filtered through a 200-mesh metal screen, centrifuged at 2000× *g* for 10 min. The cell precipitates were re-suspended in a complete endothelial culture medium, then inoculated in 2% gelation-coated culture plates, followed by incubation at 37 °C with 5% CO_2_ for 24 h. The nonadherent cells were washed off with DMEM and the adherent cells were incubated with a complete endothelial culture medium at 37 °C with 5% CO_2_ until the cells grew to nearly 80% confluence. The magnetic beads coated with Ulex europaeus agglutinin I were added into the cell culture well, then we added 1 mL of 0.1% trypsin for digestion after the magnetic beads combined well under the microscope. The digested cell fluid was collected in a centrifuge tube, then the combined cells and uncombined cells were separated using a magnetic bead collector and collected. A fluorescence microscope (Olympus, Beijing, China) was used to detect the typical marker on the cell surface after immunofluorescence staining. The collected cells were inoculated into a 2% gelatin-coated culture flask and cultured with a complete endothelial cell culture medium (ingredients: 80% M199, 20% FBS, 2 mmol/L of glutamine, 100 U/mL of penicillin, and 100 μg/mL of endothelial cell growth factor) at 37 °C with 5% CO_2_. The passage was carried out when the monolayer cells had grown to cover 80% of the culture flask bottom area, at which point we took the third generation of cells for the next step of the experiment.

### 2.2. Extracellular Vesicle Isolation and Identification

#### 2.2.1. Generation of BMSCs from Bone Marrow of a Mouse

The mice were adopted in the generation of the MSCs. The mice femur and tibia were exposed under sterile conditions in supine fixation and rinsed twice with PBS (Procell, Wuhan, China). The bone marrow cavity was exposed and rinsed twice with 2 mL of MEMα (Procell, Wuhan, China), then the bone marrow was collected. The bone marrow was filtered through a 200-mesh cell screen and collected in a 15 mL centrifuge tube, centrifuged at 1200 rpm for 5 min. The cell precipitates were re-suspended with 4 mL of MEMα (Procell, Wuhan, China), then added into a 15 mL centrifuge tube with 4 mL of mouse bone marrow lymphocyte separation solution, centrifuged at 2000 rpm for 20 min. The intermediate white membrane cells were transferred into a 15 mL centrifuge tube, to which we added 10 mL of PBS to dilute, centrifuged at 1500 rpm for 5 min. The cell precipitates were re-suspended in a mouse MSCs complete culture medium (Procell, Wuhan, China), and the cells were inoculated with 2 × 10^6^ cells/mL in a polylysine-pre-coated petri dish, then incubated at 37 °C in 5% CO_2_ constant temperature for 3 days. The passage was carried out when the cells were fully grown, and the cell morphology was observed under a phase contrast microscope (Olympus). The second generation of cells was selected for the next step of the experiment.

#### 2.2.2. Isolation and Identification of BMSC-EVs

EVs were isolated from MSC supernatants as previously described [23,24]. The second generation of BMSCs with good growth were collected, centrifuged at 3000× *g* for 15 min to remove cellular debris, then the cell supernatant was collected after centrifugation. A total of 5 mL of cell supernatant was collected in a centrifuge tube, and exosome precipitation reagent (Rengen Bioscience, Liaoning, China) was added, mixed, and left standing at 4 °C for 30 min, then ultracentrifuged at 100,000× *g* at 4 °C for 30 min. The supernatant was removed, and the EVs’ precipitation was re-suspended with 100 μL of PBS. The re-suspended EVs were transferred into the purification column, centrifuged at 2000× *g* at 4 °C for 5 min, and then collected. Transmission electron microscopy (JEOL, Tokyo, Japan) was used to observe the morphology of the EVs. Nanoparticle tracking analysis (NTA) was used to detect the size distribution and concentration of EVs using ZetaView (Particle Metrix, Meerbusch, Germany). The protein concentration of the EVs was measured using a bicinchoninic acid (BCA) protein assay kit (Beyotime, Shanghai, China). A Western blot analysis was performed to identify surface markers of MSC-EVs, including CD9, CD63, and CD81 [25]. The images were captured.

### 2.3. Cell Treatment

#### 2.3.1. Establishment of Glucocorticoid-Induced BMECs Injury Model

The third generation of BMECs was selected and cultured. Cells in the logarithmic growth stage and in a good growth state were seeded into 24-well Matrigel (Corning, Corning, NY, USA) culture plates at a density of 5 × 10^3^ cells per well overnight. The cells in the well were added into the medium containing a series of concentration-gradient hydrocortisone amounts (0 mg/mL, 0.03 mg/mL, 0.1 mg/mL, 0.3 mg/mL and 1 mg/mL); continued culturing for 12 h, 24 h, and 48 h at 37 °C with 5% CO_2_; and then each well was incubated with 10 μL of CCK-8 solution for 4 h away from light before we measured the absorbance at 450 nm using a Thermo Varioskan LUX multimode microplate reader. The appropriate concentration of hydrocortisone will be used for the next step of the experiment.

#### 2.3.2. Administration of EVs to Glucocorticoid-Induced BMECs Injury Model

The third generation BMECs was selected and randomly divided into five groups: the control group (no special treatment), model group (treated with 0.1 mg/mL of hydrocortisone), model + EVs group(0.1 mg/mL of hydrocortisone + 400 μg/mL of exos), model + rapamycin (MedChemExpress, Monmouth Junction, NJ, USA) group (0.1 mg/mL of hydrocortisone + 50 nM of rapamycin), and model + EVs + LY294002 (MedChemExpress, Monmouth Junction, NJ, USA) group (0.1 mg/mL hydrocortisone + 400 μg/mL EVs + 25 μM LY294002). Each group had three samples.

### 2.4. Cell Migration and Invasion Ability Analysis and Capillary Network Formation Assay

The third generation of BMECs was selected and washed with 3 mL of PBS, then digested with 0.25% trypsin, centrifuged at 1000 rpm for 5 min. The precipitates were washed to remove residual serum and re-suspended in a serum-free medium. The BMECs were preconditioned as aforementioned. The cell concentration was diluted to 3 × 10^5^ cell/mL in each group. A total of 200 μL of cell suspension was plated into the upper chambers of a transwell plate (Corning), and 800μL of 10% FBS medium, placed in the lower chamber, was used as a chemoattractant. Then, the cells were cultured at 37 °C with 5% CO_2_ for 24 h. The membranes were fixed with ethanol and stained with crystal violet (Beyotime), then mounted and observed under a light microscope (Olympus), and the number of cells was counted.

A tube formation assay was performed to investigate the endothelial cell network formation. Briefly, BMECs were seeded onto Matrigel-coated 24-well plates at a density of 1 × 10^5^ cells per well. The BMECs were preconditioned as aforementioned and cultured at 37 °C with 5% CO_2_ for 24 h. Images were captured, and the number of meshes and tubule lengths was quantified by the Image J software.

### 2.5. Cell Apoptosis Analysis

Annexin V-FITC/PI kits (Elabscience, Wuhan, China) were used to assess cell apoptosis. The BMECs were preconditioned as aforementioned, then removed from the medium and washed with PBS. The BMECs were harvested using 0.25% trypsinization and transferred to Eppendorf tubes, then re-suspended with 300 μL of binding buffer, to which 5 μL of annexin V and 5 μL of PI was added. These were incubated for 10 min at room temperature in the dark and analyzed by a flow cytometer.

### 2.6. Cell Viability Assay

Cell Counting Kit-8 was used to test cell viability. Approximately 5 × 10^3^ BMECs were seeded in 96-well plates with 100 μL of medium in each well. The BMECs were preconditioned as aforementioned, then cultured at 37 °C with 5% CO_2_ for 24 h. Then, each group was incubated with 10 μL of CCK-8 solution for 4 h away from light before the absorbance was measured at 450 nm by a Thermo Varioskan LUX multimode microplate reader.

### 2.7. Western Blot Analysis

The BMECs of each group were collected and the total protein was extracted by a total protein extraction kit (Beyotime). A BCA protein assay kit (Beyotime) was used to measure the protein concentration. A 60µg sample of each group was loaded onto 10% SDS-PAGE electrophoresis, then transferred to a polyvinylidene difluoride membrane. The membrane was blocked with TBST containing 5% skim milk, followed by incubation with the primary antibodies of LC3 (Abcam, Cambridge, UK, dilution 1:1000), PI3K (Proteintech Group, Wuhan, China, dilution 1:2000), P62 (Abcam, Cambridge, UK, dilution 1:1000), p-Akt (Abcam, Cambridge, UK, dilution 1:5000), Akt (Proteintech Group, Wuhan, China, dilution 1:2000), p-mTOR (Bioss, Beijing, China, dilution 1:1000), mTOR (Bioss, Beijing, China, dilution 1:1000), and GADPH (Hangzhou Goodhere Biotechnology, China, dilution 1:1000) at 4 °C overnight, then washed with TBST for 10 min and incubated with secondary antibodies for 2 h at room temperature.

Finally, the result was visualized by a chemiluminescence detection system (FluorChem M, ProteinSimple, San Jose, CA, USA).

### 2.8. Immunofluorescence Analysis

The BMECs were grown on glass coverslips in 24-well plates. After being treated by group, the BMECs were fixed with 3.7% paraformaldehyde at 37 °C for 30 min, and then made into frozen slices with a thickness of 5 μm after sucrose gradient dehydration. The BMECs were penetrated with 0.25% TritonX-100 at 37 °C for 30 min, and blocked BMECs with 10% goat serum in PBST (PBS + 0.1% Tween 20) for 30 min. The BMECs were incubated in the primary antibody of LC3 (Abcam; dilution 1:200) in a humidified chamber for 1 h at room temperature. The cells were then incubated with the secondary antibody for 1 h at room temperature in the dark after being washed three times for 5 min with PBS. Then, we decanted the secondary antibody solution and washed the cells three times for 5 min with PBS in the dark. Finally, we mounted the coverslip with a drop of mounting medium with 4′6-diamidino-2-phenylindole (DAPI). All imaging analyses were performed using fluorescence microscopy (Olympus).

### 2.9. Transmission Electron Microscopy

The BMECs were grown on glass coverslips in 24-well plates. After being treated by group, BMECs were washed with a phosphoric acid buffer, and then fixed with 1% osmium acid (Pelco) at 4 °C for 3 h. The BMECs were washed three times with the buffer, then dehydrated with ethanol, replaced by propylene oxide, and then polymerized in an oven at 70 °C. The samples were observed and photographed under a transmission electron microscope (JEM1230) after being sliced by the ultrathin slicer (EM UC6) and stained with uranium dioxy acetate (Spi-Chem) and lead citrate (Spi-Chem).

## 3. Statistical Analysis

All data are shown as mean ± standard deviation (SD). Differences between groups were assessed by one-way analysis of variance (ANOVA). Statistical analyses were performed using SPSS 18.0 software (SPSS, Inc., Chicago, IL, USA). *p* values < 0.05 were considered statistically significant.

## 4. Results

### 4.1. Isolation, Purification, and Culture of Microvascular Endothelial Cells

**Figure 1:**
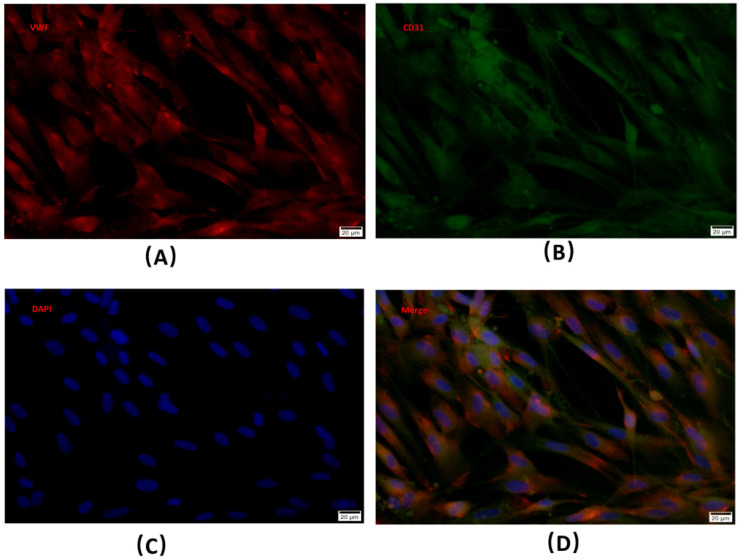
(**A**–**D**) Identification of microvascular endothelial cells. BMECs-specific markers were detected via immunofluorescence staining.

The BMECs were successfully isolated from a rabbit femoral head by using magnetic beads, and then being cultured. Immunofluorescence staining showed that the cells expressed typical marker molecules vWF and CD31 of endothelial cells (Figure 1A–D).

### 4.2. Characterization of BMSC-EVs

**Figure 2:**
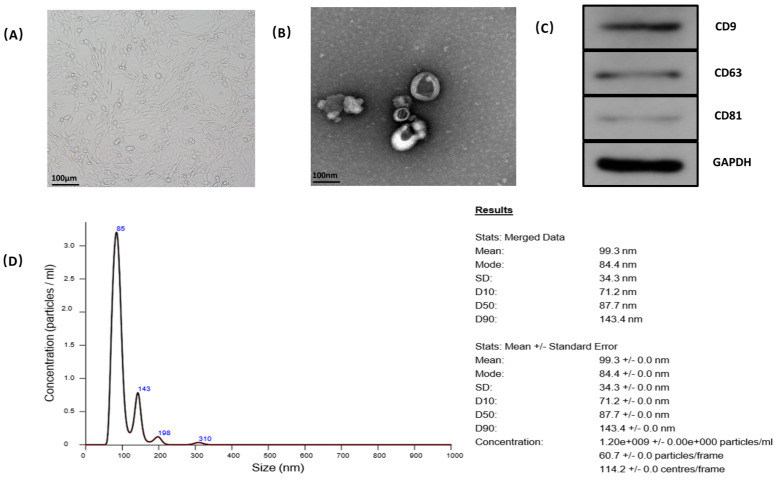
Characterization of BMSC-EVs. (**A**) The fibroblast-like morphology of BMSCs shown by microscope. (**B**) The morphology of BMSC-EVs shown by scanning electron microscopy. (**C**) Expression of CD9, CD63, and CD81 incorporation into BMSC-EVs shown by Western blotting. (**D**) Identification of size and concentration of BMSC by nanoparticle tracking analysis.

The BMSCs were successfully isolated and cultured from the mouse. The cells displayed a homogeneous fibroblastic-like morphology (Figure 2A). SEM, Western blotting and dynamic light scattering were used to characterize the purified particles derived from MSCs. SEM images showed that EVs exhibited spheroidal morphology (Figure 2B). The results of Western blotting confirmed the expression of CD9, CD63, and CD81 in EVs, which are surface markers exceptionally enriched in EVs (Figure 2C). The protein concentration of EVs was 4.63 mg/mL, tested by BCA. NTA revealed that the average diameter was 99.3 nm with a mean concentration of 1.2 × 10^9^ particles/mL, and the number of exosomes with the diameter of 84.4 nm was the largest (Figure 2D).

### 4.3. Appropriate Concentration of Glucocorticoid-Damaging BMECs

**Figure 3:**
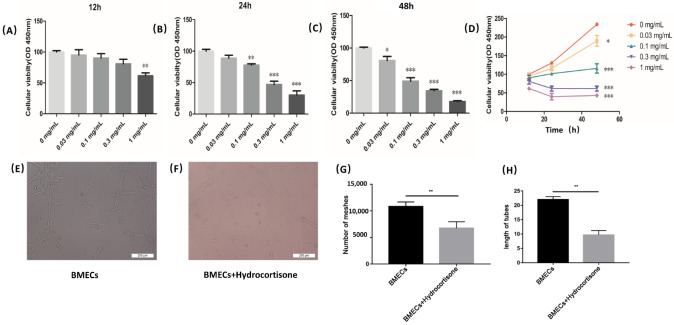
Hydrocortisone decreased BMECs’ viability. (**A**–**D**) CCK-8 was used to measure viability of BMECs treated with different concentrations of hydrocortisone for different times. (**E**–**H**) When 0.1 mg/mL of hydrocortisone was used to treat BMECs, the number of meshes and tube length of BMECs were significantly reduced, indicating that BMECs injury model was successfully established. OD value; * *p* < 0.05, ** *p* < 0.01, *** *p* < 0.005. Each bar represents the mean ± SD of three independent experiments.

BMECs were treated with different concentrations of gradient hydrocortisone. Cell Counting Kit-8 was used to test the cell viability (Figure 3A–D). The indication of the viability of BMECs was a decreased tendency as the concentration of hydrocortisone increased and treating time was extended. The 0.1 mg/mL of hydrocortisone was selected in the subsequent experiments as the appropriate concentration (Figure 3E–H).

### 4.4. The Influence of BMSC-EVs on Glucocorticoid-Induced BMECs Injury

#### 4.4.1. BMSC-Derived EVs Promote Migration, Invasion Capacity, and Angiogenesis of BMECs

**Figure 4:**
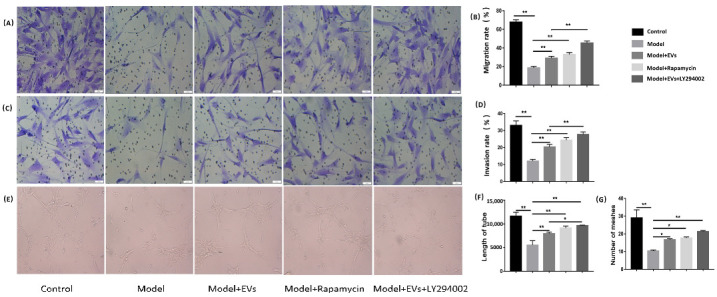
Angiogenesis was promoted by BMSC-EVs. (**A**) The migration capacity of BMECs was investigated by transwell assay in different groups. (**B**) Quantitative analysis of the migration rate of BMECs. (**C**) The invasion capacity of BMECs was investigated by transwell assay in different groups. (**D**) Quantitative analysis of the invasion rate of BMECs. (**E**) Tube formation assay for detecting the tube-forming ability of BMECs in the different groups. (**F**,**G**) Quantitative analysis of tube formation. The value of the total mesh area, total length was measured. Each bar represents the mean ± SD of three independent experiments. * *p* < 0.05, ** *p* < 0.01.

In a transwell assay, hydrocortisone significantly decreased the migration and invasion capacity of BMECs compared to the control group. Compared with the model group, the inhibited effect was reversed after being treated with the BMSC-EVs. Simultaneously, the inhibition of migration and invasion capacity of BMECs was reversed with the administration of rapamycin. Compared with the Model + EVs group, the migration and invasion capacity of BMECs improved further after adding LY294002 (Figure 4A–D). In the tube formation assay, the model group showed a significant antiangiogenic manifestation compared with control group. BMSC-EVs, rapamycin and LY294002 reversed the inhibitory effect of angiogenesis and increased the loop formation ability of BMECs (Figure 4E). The number of meshes and lengths of tubes increased after adding BMSC-EVs, rapamycin, and LY294002 (Figure 4F). These results showed that BMSC-EVs can promote angiogenesis.

#### 4.4.2. BMSC-Derived EVs Prevented Glucocorticoid-Induced Apoptosis of BMECs

**Figure 5:**
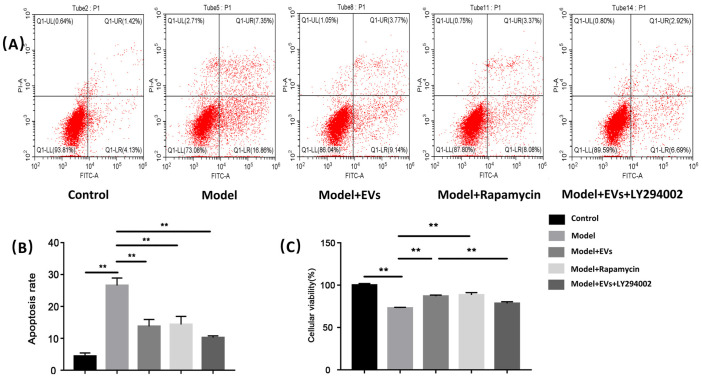
BMSC-EV-derived exosomes BMECs’ viability and protected BMECs against glucocorticoid-induced apoptosis. (**A**) Apoptosis was quantified by flow cytometry after staining with annexin V-FITC/PI. (**B**) Percentage of apoptotic cells in different groups. (**C**) CCK-8 was used to measure viability of BMECs in different groups. Each bar represents the mean ± SD of three independent experiments. ** *p* < 0.01.

Flow cytometry demonstrated that the percentage of apoptotic cells increased from 4.42% (control group) to 26.60% (model group) after treatment with hydrocortisone (*p* < 0.01). MSCs-EVs showed a protective effect on hydrocortisone-induced apoptosis of BMECs. When MSCs-EVs were added, the percentage of apoptotic cells decreased from 26.60% (model group) to 13.76% (model + exos group) (*p* < 0.01). When rapamycin was added, the percentage of apoptotic cells decreased from 26.60% (model group) to 14.34% (model + rapamycin group) (*p* < 0.01), and its effect was similar to the effect caused by BMSC-EVs. When the PI3K inhibitor LY294002 was added, the percentage of apoptotic cells further decreased from 13.76% (model + EVs group) to 7.36% (model + EVs + LY294002 group) (Figure 5A,B).

#### 4.4.3. BMSC-Derived EVs Alleviate the Decreased Cell Viability of BMECs Induced by GCs

Cell Counting Kit-8 was used to test the cell viability. Compared with the control group, the cell viability significantly decreased after being treated with hydrocortisone. Compared with the model group, the cell viability significantly increased after being treated with BMSC-EVs, indicating that BMSC-EVs effectively alleviate glucocorticoid-induced BMEC injury. Meanwhile, the addition of rapamycin as an autophagy activator can effectively alleviate the decreased viability caused by hydrocortisone, suggesting rapamycin alleviated the injury of BMECs caused by hydrocortisone by activating autophagy. Compared with the model + EVs group, the treatment with LY294002 as the PI3K inhibitor effectively inhibited the alleviating effect of BMEC-EVs on glucocorticoid-induced injury, suggesting that BMSC-EVs alleviated hydrocortisone-induced injury mainly through the activation of autophagy (Figure 5C).

#### 4.4.4. BMSC-EVs Regulated Autophagy of BMECs

**Figure 6:**
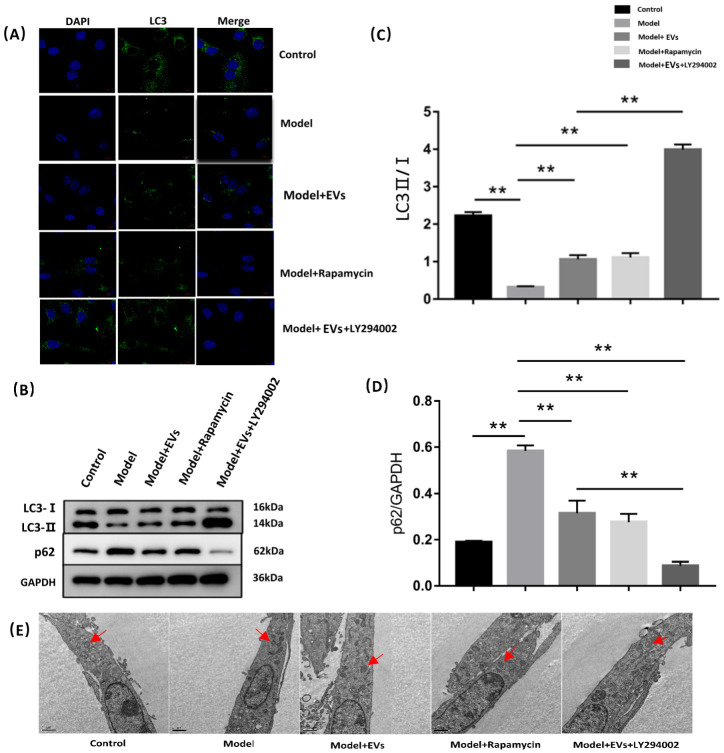
BMSC-EVs regulated autophagy of BMECs. (**A**) Immunohistochemical analysis of autophagic marker LC3. Scale bar = 10 μm. (**B**) Western blot analysis of LC3 and P62 in different groups. (**C**,**D**) Quantification of LC3 and P62 in different groups. (**E**) Morphological changes of autophagosomes were observed by TEM in different groups and the red arrow represents the autophagosome location. Each bar represents the mean ± SD of three independent experiments. ** *p* < 0.01. DAPI, 4′,6-diamidino-2-phenylindole; EVs, BMSC-derived extracellular vesicles; GAPDH, glyceraldehyde 3-phosphate dehydrogenase; mTOR, mammalian target of rapamycin.

LY294002 was used as an autophagy inhibitor, and rapamycin was used as an autophagy inducer to observe the effects of BMSC-derived exosomes on cell autophagy. Western blot analysis revealed that hydrocortisone significantly decreased the ratio of LC3-II/I, and increased the expression of P62 (an autophagy protein receptor) (Figure 6B–D). Accordingly, immunofluorescence analysis showed the number and intensity of punctate LC3 fluorescence decreased in the model group. Compared with the model group, the ratio of LC3-II/I was upregulated, and the expression of p62 was downregulated in the model + EVs group, and its effect was similar to the model + rapamycin group. When LY294002 was added, the ratio of LC3-II/I was further upregulated and the expression of p62 was further downregulated. Moreover, the number and intensity of punctate LC3 fluorescence increased in the model + EVs group, and it was more than the model + rapamycin group, but less than the model + EVs + LY294002 group (Figure 6A).

Transmission electron microscopy was used to observe the morphology. Compared with the control group, hydrocortisone made the autophagosome fold and shrink and reduce its number. The morphology of the autophagosome in the model + EVs group was similar to that of the control group, while the change of the autophagosome in the model + EVs + LY294002 group was not significant compared with the model + EVs group (Figure 6E).

These results suggested that BMSC-EVs can promote autophagy of BMECs, and the inhibition of the PI3K signal further increased the level of autophagy.

#### 4.4.5. BMSC-Derived EVs Prevent Glucocorticoid-Induced BMECs Injury by Regulating Autophagy via the PI3K/Akt/mTOR Pathway

**Figure 7:**
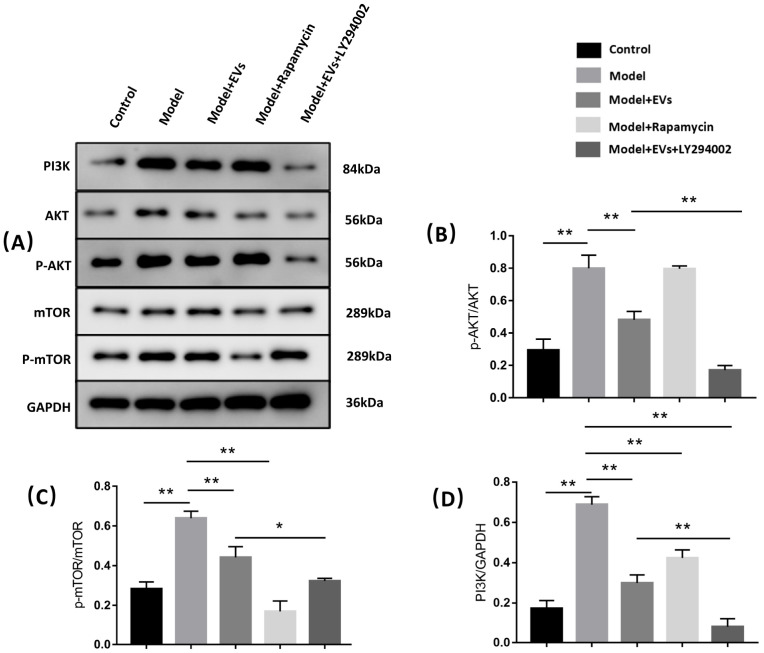
BMSC-EVs regulated the level of BMECs autophagy via PI3K/Akt/mTOR pathway. (**A**) Western blot analysis. (**B**–**D**) Quantification of p-Akt, Akt, p-mTOR, mTOR and PI3K in five groups. Each bar represents the mean ± SD of three independent experiments. * *p* < 0.05, ** *p* < 0.01. EVs, BMSC-derived extracellular vesicles; GAPDH, glyceraldehyde 3-phosphate dehydrogenase; mTOR, mammalian target of rapamycin.

To assess whether BMSC-EVs prevent glucocorticoid-induced BMEC injury by regulating autophagy via the PI3K/Akt/mTOR pathway, LY294002 (an inhibitor of PI3K) and rapamycin (an inhibitor of mTOR) were used. As shown in Figure 7, hydrocortisone induced a significant upregulation of PI3K, p-mTOR/mTOR, and p-Akt/Akt expression, and this effect could be reversed by treatment with BMSC-EVs (Figure 7A–D). When rapamycin was added, the level of p-mTOR/mTOR and PI3K significantly downregulated, but the level of p-Akt/Akt slightly upregulated. Compared with the model + EVs group, the expression of p-mTOR/mTOR, p-Akt/Akt, and PI3K downregulated in the model + EVs + Y294002 group (Figure 7A–D).

## 5. Discussion

Long-term or short-term overuse of GCs can cause local ischemia and secondary osteonecrosis, which are considered core pathological mechanisms in ONFH. BMEC is an important part of bone microcirculation, and BMEC injury caused by GCs is an important mechanism of ONFH [26]. The mechanism of injury caused by GCs to BMECs is not clear. Sbardella et al. found that dexamethasone inhibited trabecular meshwork cells by downregulating autophagy, a kind of secretory endothelial cell similar to BMECs, causing metabolic disorder and even apoptosis [27]. Under normal circumstances, autophagy is at a low level, and only moderate autophagy has a protective effect. Inappropriate autophagy will inhibit the normal function of autophagy, leading to apoptosis and injury to the cell [28,29]. Meanwhile, Liu et al. reported that extracellular vesicles secreted from human-induced pluripotent stem cells improved migration and the tube-formation ability of human umbilical vascular endothelial cells [16]. The in vitro results suggested BMSC-Exos and autophagy might be associated with the progression of steroid-induced ONFH.

Stem cell transplantation has been shown to improve local blood to ONFH by releasing various cytokines to promote angiogenesis [30]. However, the potential risk and problems are the major limitation for the possibility of clinical application. EVs derived from MSCs are studied extensively and demonstrate equal efficacy as MSCs [31]. Previous studies have identified that transplantation of exosomes derived from MSCs did not induce an immune reaction in vivo [20,32]. EVs secreted by stem cells have been demonstrated to have a similar effect to promote angiogenesis [22]. Liu et al. reported that MSC-derived exosomes inhibited H9C2 cell apoptosis induced by hypoxia and serum deprivation, and improved H9C2 cell viability by reducing excess autophagy activity via activation of the PI3K/Akt/mTOR pathway [33]. The extraction of EVs was performed by a combination of ultrafiltration and purification. The obtained EVs exhibited round-shaped morphology with an average diameter of 99.3 nm, and expressed typical EV surface markers including CD9, CD63, and CD81, with a mean concentration of 1.2 × 10^9^ particles/mL.

The potential mechanism of BMSC-EVs preventing glucocorticoid-induced BMEC injury was also investigated in this study. The most important function of EVs is their role in communication from host cells to target cells. EVs transport a variety of proteins, lipids, RNA, and other substances to the site of injury, and play an important role in angiogenesis, anti-apoptosis, and anti-inflammatory responses [34]. We found that BMSC-EVs could alleviate the decreased cell viability induced by glucocorticoids, improve migration, invasion capacity, and tube formation, and prevent glucocorticoid-induced apoptosis of BMECs. We further found that BMSC-EVs upregulated the expression of LC3 and downregulated the expression of P62, and reversed the glucocorticoid-induced folding and contraction of autophagosome. This illustrated that BMSC-EVs inhibited glucocorticoid-induced BMEC apoptosis, improved cell viability, and promoted angiogenesis via regulation of autophagy activity. Interactions between autophagy and apoptotic components suggested complex crosstalk. mTOR, a serine/threonine kinase, can promote cell growth and inhibit autophagy via various pathways, and is also an important downstream target of Akt, which plays a critical role in regulating apoptosis [13,35]. Hsu et al. demonstrated that hyperphosphatemia-induced protective autophagy in endothelial cells through the inhibition of Akt/mTOR signaling and inhibited high-Pi-induced autophagy aggravates endothelial cell apoptosis [36]. Our results showed that GCs could activate the PI3K/Akt/mTOR pathway, inhibit BMEC viability, angiogenesis, and cause cell apoptosis. However, when the pathway was blocked by the PI3K inhibitor and mTOR inhibitor, the detrimental effect elicited by glucocorticoids was alleviated. When BMSC-EVs were added, the detrimental effect elicited by GCs was also alleviated. These results suggest that GCs can inhibit autophagy by activating the PI3K/Akt/mTOR pathway, causing a decrease in BMEC viability, migration, invasion and tube-forming capacity, and even apoptosis. BMSC-EVs can prevent glucocorticoid-induced BMEC injury by regulating autophagy via the PI3K/Akt/mTOR pathway. Our experiment also has some limitations: first, we did not use filters to isolate BMSC-EVs; second, we did not detect negative markers of EVs; third, we did not use AngioTool software in the tube formation assay. We will further improve our experimental method in future experiments.

## 6. Conclusions

We identified that GCs inhibit autophagy by activating the PI3K/Akt/mTOR pathway, causing a decrease in BMEC viability, angiogenesis capacity, and even apoptosis. BMSC-EVs can regulate autophagy via the PI3K/Akt/mTOR pathway of BMECs to enhance cell viability and angiogenesis capacity. Our findings suggest that BMSC-EVs may be a promising therapeutic approach in the treatment of ONFH.

## Data Availability

All data generated or analyzed during this study are included in this published article and are available from the corresponding author upon reasonable request.
