# Correction: Ma et al. Extracellular Vesicles from BMSCs Prevent Glucocorticoid-Induced BMECs Injury by Regulating Autophagy via the PI3K/Akt/mTOR Pathway. *Cells* 2022, *11*, 2104

**DOI:** 10.3390/cells13141167

**Published:** 2024-07-09

**Authors:** Jinhui Ma, Mengran Shen, Debo Yue, Weiguo Wang, Fuqiang Gao, Bailiang Wang

**Affiliations:** 1 Department of Orthopaedic Surgery, Center for Osteonecrosis and Joint Preserving & Reconstruction, China-Japan Friendship Hospital, Beijing 100029, China; majinhui@zryhyy.com.cn (J.M.); yuedebo@zryhyy.com.cn (D.Y.); wangweiguo@zryhyy.com.cn (W.W.); 2 Department of Orthopaedic Surgery, Peking University China-Japan Friendship School of Clinical Medicine, Beijing 100029, China; 2111210577@stu.pku.edu.cn

In the original publication [1], there was a mistake in Figure 5A as published. The panels for Model+EVs and Model+EVs+LY294002 were the same. The corrected Figure 5A appears below. The authors state that the scientific conclusions are unaffected. This correction was approved by the Academic Editor. The original publication has also been updated.

## Figures and Tables

**Figure 5:**
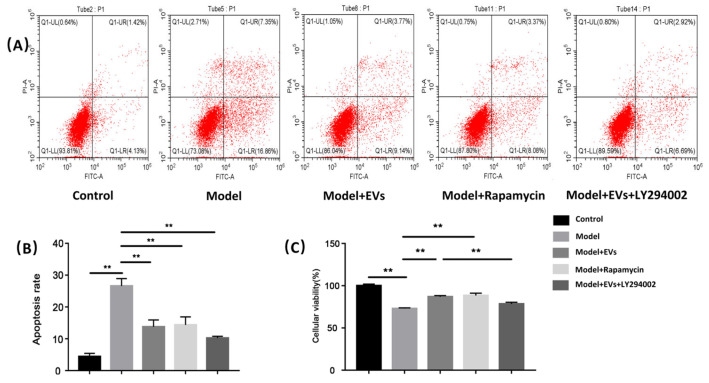
BMSC-EV-derived exosomes BMECs’ viability and protected BMECs against glucocorticoid-induced apoptosis. (**A**) Apoptosis was quantified by flow cytometry after staining with annexin V-FITC/PI. (**B**) Percentage of apoptotic cells in different groups. (**C**) CCK-8 was used to measure viability of BMECs in different groups. Each bar represents the mean ± SD of three independent experiments. ** *p* < 0.01.
